# Decreased Immunity to Varicella Zoster Virus in Giant Cell Arteritis

**DOI:** 10.3389/fimmu.2017.01377

**Published:** 2017-10-24

**Authors:** Christien Rondaan, Kornelis S. M. van der Geest, Elisabeth Eelsing, Annemieke M. H. Boots, Nicolaas A. Bos, Johanna Westra, Elisabeth Brouwer

**Affiliations:** ^1^Department of Rheumatology and Clinical Immunology, University of Groningen, University Medical Centre Groningen, Groningen, Netherlands

**Keywords:** giant cell arteritis, polymyalgia rheumatica, varicella zoster virus, herpes zoster, cell-mediated immunity, humoral immunity, viral immunity, glucocorticoids

## Abstract

**Introduction:**

Herpes zoster, which can have a major impact on quality of life, results from reactivation of a latent varicella zoster virus (VZV) infection. We hypothesized that giant cell arteritis (GCA) patients are at increased risk of herpes zoster because of treatment with high-dose glucocorticoids and advanced age. Aim of the study, therefore, was to determine cell-mediated and humoral immunity to VZV in patients with GCA, patients with closely related disease polymyalgia rheumatica (PMR; treated with lower doses of glucocorticoids) and healthy controls (HCs).

**Methods:**

Cell-mediated immunity to VZV was determined by performing interferon-γ (IFNγ) enzyme-linked immunospot and intracellular cytokine flow cytometry measurements in 11 GCA and 15 PMR patients and in 26 age/sex-matched HCs. Immunoglobulin G antibodies to VZV glycoprotein (VZV-IgG) were measured in serum samples of 35 GCA and 26 PMR patients at different times of follow-up and in 58 age and sex-matched HCs by an enzyme-linked immunosorbent assay.

**Results:**

The number of VZV-specific IFNγ spot-forming cells was significantly lower in GCA patients on treatment, than in age-matched HCs (*p* = 0.029), but was not different in PMR patients on treatment. Similar levels of VZV-IgG were found in GCA and PMR patients at baseline, compared to HCs.

**Conclusion:**

The finding of a decreased cell-mediated immunity to VZV, known to be of great importance in defense to the virus, indicates an increased herpes zoster risk in GCA patients compared to an already at-risk elderly population. Herpes zoster vaccination is, therefore, of special importance in GCA patients, and would ideally be administered at time of diagnosis. Interestingly, as VZV was suggested to be the trigger in GCA pathogenesis, similar levels of VZV-IgG were found in GCA patients at time of diagnosis and age-matched HCs, indicating that GCA patients did not experience herpes zoster substantially more often in the months preceding diagnosis than controls.

## Introduction

Herpes zoster is caused by reactivation of a latent varicella zoster virus (VZV) infection (1). Almost the whole adult population of temperate countries has experienced a primary VZV infection in childhood (known as varicella or chickenpox) and is, therefore, at risk of herpes zoster (2). Postherpetic neuralgia (pain lasting >90 days after onset of rash) is the most common complication of herpes zoster occuring in 8–27% of patients (3–6). Impact on quality of life of herpes zoster and postherpetic neuralgia can be major (3, 4).

With advancing age, a rise in the incidence of herpes zoster occurs concomitantly with a decline in VZV-specific cell-mediated immunity. The estimated incidence of HZ is about 3.4–4.82 per 1,000 person years in the general population which increases to more than 11 per 1,000 person years in those above 80 years of age (7).

Giant cell arteritis (GCA) and the closely related polymyalgia rheumatica (PMR) are inflammatory rheumatic disorders that occur almost exclusively in older adults (8). In our hospital, GCA patients usually start on 40–60 mg of prednisolone daily, while PMR patients start at a lower dose of 15–20 mg prednisolone daily. In both patient groups, the prednisolone dose is slowly tapered. Use of glucocorticoids is associated with a higher herpes zoster risk (9–14). Both because of advanced age and high-dose glucocorticoid use, especially GCA patients could be expected to have an increased herpes zoster risk.

A live attenuated herpes zoster vaccine was licensed in the US in 2006 for use in immunocompetent persons above 60 years of age (15). In this group, it was shown to be safe and effective in preventing herpes zoster and postherpetic neuralgia (6). The Advisory Committee on Immunization Practices (ACIP) recommends the zoster vaccine for all persons aged ≥60 years (16). Like immunocompetent older persons, GCA and PMR patients may benefit from zoster vaccination.

The ACIP in 2008, however, also stated that the vaccine should not be administered to persons on immunosuppressive therapy, including high-dose glucocorticoids (≥20 mg/day of prednisone or equivalent) lasting two or more weeks, although this recommendation was based on expert opinion only (16). The 2010 European League Against Rheumatism (EULAR) recommendations for vaccination in patients with rheumatic diseases are inconclusive, stating that zoster vaccination may be considered in patients with rheumatic diseases (17).

Since the time of ACIP and EULAR recommendations, a large retrospective cohort study in patients with immune-mediated diseases, including patients using oral glucocorticoids and even biologicals, has shown that zoster vaccination is associated with a reduced incidence of herpes zoster, also within 42 days of vaccination (a safety concern, as the vaccine is live attenuated) (10). A randomized controlled trial in patients using glucocorticoids (with a limited number of included patients using more than 10 mg daily equivalent of prednisone), comparing live attenuated zoster vaccine to placebo vaccine, demonstrated that zoster vaccination was well tolerated (18).

When balancing pros and cons of zoster vaccination in GCA patients, information on the pre-existing cellular and humoral immunity to VZV is essential. The aim of this study, therefore, was to determine both cell-mediated immunity (most important in the defense to the virus) and humoral immunity to VZV in patients with GCA, the closely related PMR and age/sex-matched healthy controls (HCs).

## Materials and Methods

### Study Population and Procedures

Prospectively collected serum samples and peripheral blood mononuclear cells (PBMCs) from patients with GCA and PMR, and matched HCs were used. Study procedures have been described previously (19, 20).

Giant cell arteritis patients either had a positive temporal artery biopsy and/or positive ^18^F-fluorodeoxyglucose-positron emission tomography-computed tomography (FDG PET-CT). PMR patients fulfilled the Chuang/Hunder criteria or had a FDG PET-CT positive scan for PMR (21).

Serum was stored at −20°C, and PBMCs were stored in liquid nitrogen until use. After evaluating cell viability by trypan blue staining, PBMCs were used in enzyme-linked immunospot (ELISpot) assays and flow cytometry analyses.

Samples for humoral analyses were randomly selected, and samples from patients at different times of follow-up were used: *T* = 0, *T* ≤ 2 months, *T* = approximately 6 months, *T* = approximately 1 year, and *T* = approximately 2 years, or more. At *T* = 0, patients had not yet received treatment with prednisolone (or other immunosuppressive drugs). At *T* ≤ 2 months, patients were within 2 months of follow-up and had all started prednisolone treatment.

Medical records from both patients and HCs were checked for a history of herpes zoster within 2 years before disease onset or sample collection. In The Netherlands, zoster vaccination is not advised and costs are not compensated in the national health-care system. Since all patients were Dutch, it could safely be presumed that none of the study subjects had been vaccinated against herpes zoster.

The study was approved by the institutional review board of the University Medical Centre Groningen (METc2012/375 for HCs and METc2010/222 for GCA and PMR patients), and written informed consent was obtained from all study participants.

### Interferon-γ (IFNγ) ELISpot Assay

Interferon-γ (IFNγ) ELISpot assay was performed as described previously (22). PBMCs were stimulated for 48 h with ultraviolet (UV)-inactivated varicella vaccine (Provarivax; Sanofi Pasteur). PBMCs stimulated with concanavalin A were used as controls. A negative control consisted of PBMCs in culture medium alone. After staining, spots were counted using an automated reader (AID EliSpot Reader; Autoimmun Diagnostika GmbH). The mean number of spots in the negative control sample was subtracted from the mean number of spots in the VZV-stimulated wells. Results are referred to as the number of IFNγ spot-forming cells per 2 × 10^5^ PBMCs.

### Intracellular Cytokine Staining Flow Cytometry

Fluorescent T cell barcoding staining for tumor necrosis factor alpha (TNFα), interleukin-2 (IL-2), and IFNγ was performed as described previously (23) after stimulating PBMCs using UV-inactivated varicella vaccine. PBMCs stimulated with staphylococcal enterotoxin B (SEB; Sigma-Aldrich) were used as positive controls. A negative control consisted of PBMCs in medium alone. PBMCs were stimulated for 18 h, of which the last 16 h in the presence of 10 µg/ml brefeldin A (Sigma-Aldrich). Kaluza software (Beckman Coulter) was used for analyses. Percentages of antigen-specific cells were expressed as the percentage of CD69+ cytokine-producing CD4+ or CD8+ T cells.

### Antibody Response to VZV

For quantitative detection of VZV-IgG antibodies, an in-house glycoprotein enzyme-linked immunosorbent assay was previously developed and validated (22). VZV purified glycoproteins (EastCoastBio) were used as antigen, and pooled human serum with known levels of anti-glycoprotein VZV was used as standard.

### Statistical Analysis

Data were analyzed using SPSS 23 (IBM) and graphs were made using GraphPad Prism 5.0 (GraphPad Software). For correlations Spearman’s rho was used. To compare differences between either the GCA or the PMR group to the HC group, Mann–Whitney *U* and Fisher’s exact test were used as appropriate. Humoral immunity results at baseline were compared to the HC group separately for GCA and PMR patients using Mann–Whitney *U* tests. *p*-Values less than 0.05 (2-tailed) were considered significant.

## Results

### Characteristics of Patients and HCs

Characteristics of patients and controls are summarized in Table 1. Levels of VZV-IgG antibodies were measured in a larger number of samples, on different time points of follow-up. Patient and control characteristics are, therefore, separately summarized in Table S1 in Supplementary Material. All patients started with prednisolone and some relapsing patients used methotrexate or leflunomide as glucocorticoid sparing drug. No other immunosuppressive medication was used.

**Table 1:** Characteristics of patients and healthy controls (HCs).

	HCs (*n* = 26)	GCA patients (*n* = 11)	PMR patients (*n* = 15)
Sex, no. female/male	19/7	8/3	10/5
Age at sample date, median (interquartile range) years	73.2 (63.2–76.9)	73.2 (67.0–74.8)	71.3 (63.3–78.2)
GCA diagnosis: FDG PET-CT|TAB|FDG PET-CT + TAB	NR	6|3|2	NR
PMR diagnosis: FDG PET-CT|Chuang|FDG PET-CT + Chuang	NR	0|0|2	1|4|10
Time since diagnosis, median (interquartile range) months	NR	8.1 (4.9–9.1)	9.1 (8.0–10.0)
Cumulative prednisolone dosage, median (interquartile range) mg	NR	5,843 (5,028–8,239)	3,385 (2,413–5,320)
Use of immunosuppressants other than prednisolone, no. (%)	NR	2 (18)^a^	2 (13)^b^
No. of patients that experienced relapse since diagnosis (%)	NR	1 (9)	4 (27)
Leukocyte count, median (interquartile range) 10^9^ cells/L	5.50 (5.00–6.53)	9.80 (9.45–12.38)***	8.30 (7.30–10.90)***
Lymphocyte count, median (interquartile range) 10^9^ cells/L	1.75 (1.50–1.92)	1.68 (1.43–2.55)	1.37 (1.08–1.61)***
CD3+ lymphocyte count, median (interquartile range) 10^9^ cells/L	1.24 (1.08–1.36)	1.30 (1.06–1.85)	1.06 (0.81–1.14)*
Hemoglobin, median (interquartile range) mmol/L	8.7 (8.2–9.2)	7.9 (6.6–8.3)**	8.3 (7.5–9.3)
ESR, median (interquartile range) mm/h	9 (4–19)	24 (14–42)*	13 (8–38)
CRP, median (interquartile range) mg/L	<5 (<5–<5)	6 (<5–24)**	6.4 (<5–13)***

*GCA, giant cell arteritis; PMR, polymyalgia rheumatica; FDG-PET/CT, no. of patients with ^18^F-fluorodeoxyglucose-positron emission tomography-computed tomography supporting diagnosis; TAB, no. of patients with temporal artery biopsy positive for GCA; NR, not reported; Chuang, no. of patients fulfilling the Chuang and Hunder PMR criteria; ESR, erythrocyte sedimentation rate; CRP, C-reactive protein*.

*^a^Two patients received methotrexate (both 15 mg/week)*.

*^b^One patient received methotrexate (15 mg/week) and one patient received leflunomide (10 mg/day)*.

*p-Value < 0.05; **p-value < 0.01; ***p-value < 0.001 for comparison to HC group.

### Cellular Immunity to VZV: IFNγ ELISpot Assay

**Figure 1:**
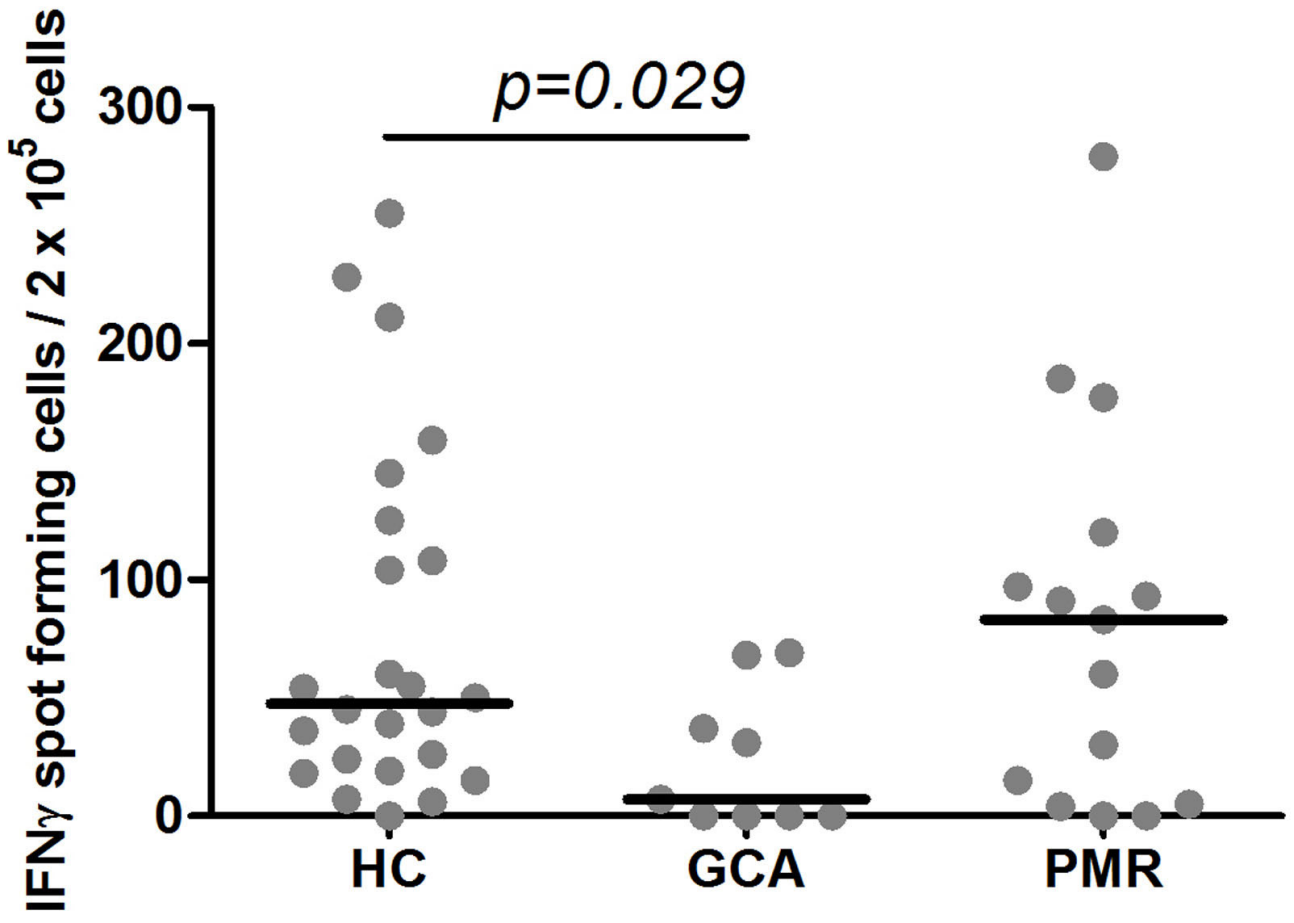
Levels of interferon-γ (IFNγ) spot-forming cells in response to varicella zoster virus stimulation. Numbers of IFNγ spot-forming cells per 2 × 10^5^ peripheral blood mononuclear cells in 24 healthy controls (HC) subjects, 9 giant cell arteritis (GCA) patients, 15 polymyalgia rheumatica (PMR) patients are shown. Results are corrected for responses in non-stimulated cultures from the same sample. Bars show the median.

The number of IFNγ spot-forming cells was significantly lower among GCA patients on treatment as compared to HCs (*p* = 0.029). PMR patients on treatment and HCs showed similar numbers of spot-forming cells. The median number of IFNγ spot-forming cells per 2 × 10^5^ PBMCs in response to VZV stimulation was 47.5 (range 0–255) among HCs, 7 (range 0–69) among GCA patients, and 83 (range 0–279) in PMR patients (Figure 1).

### Cellular Immunity to VZV: Flow Cytometric Analysis of Cytokine Production

**Figure 2:**
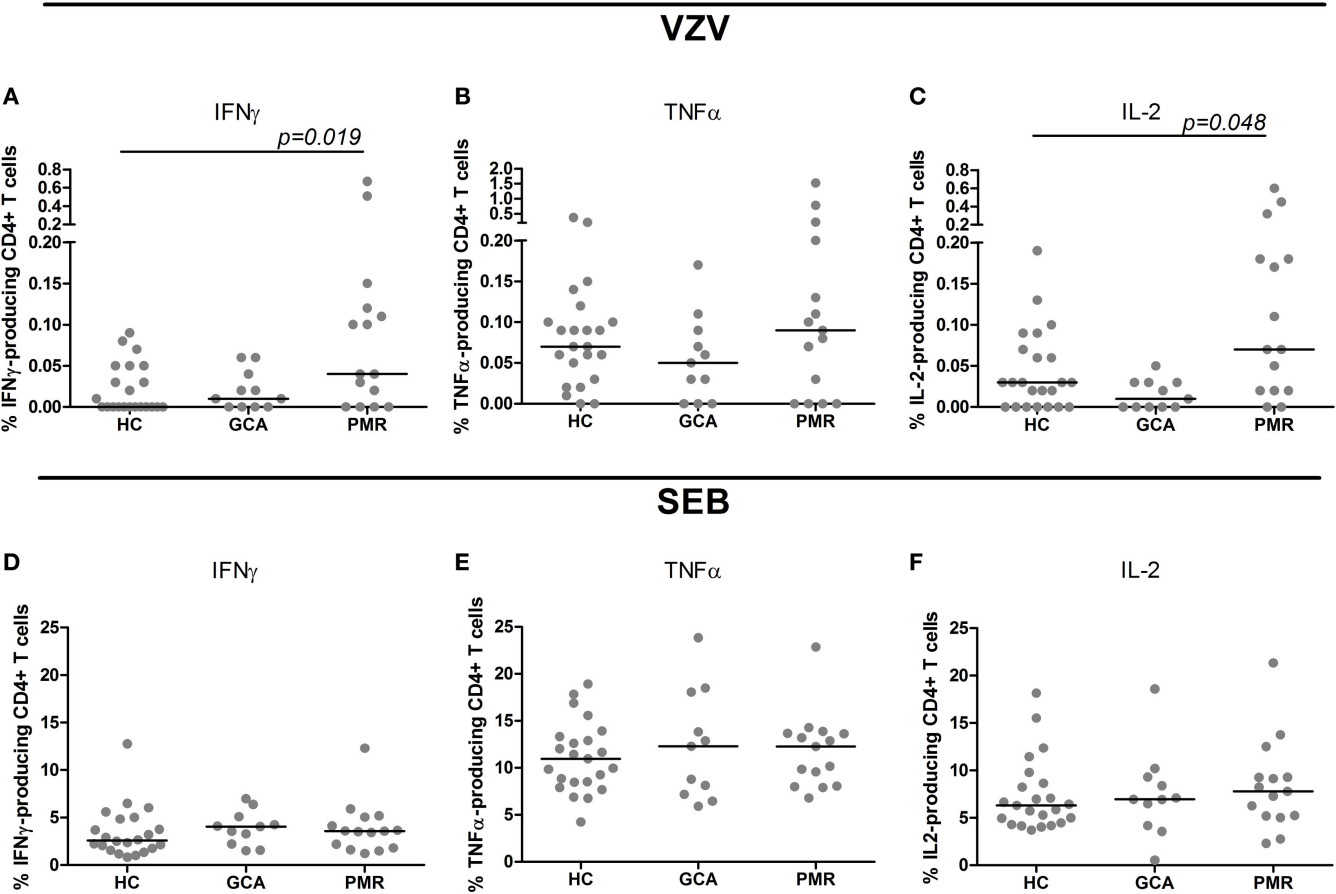
Frequencies of cytokine-producing CD4+ T cells upon stimulation with varicella zoster virus (VZV) **(A–C)** and staphylococcal enterotoxin B (SEB, positive control) **(D–F)** in healthy control (HC) subjects (*n* = 23), giant cell arteritis (GCA) patients (*n* = 11) and polymyalgia rheumatica (PMR) patients (*n* = 15). Results are corrected for responses in non-stimulated cultures. Bars show the median. IFNγ, interferon-γ; TNFα, tumor necrosis factor α; IL-2, interleukin-2.

**Figure 3:**
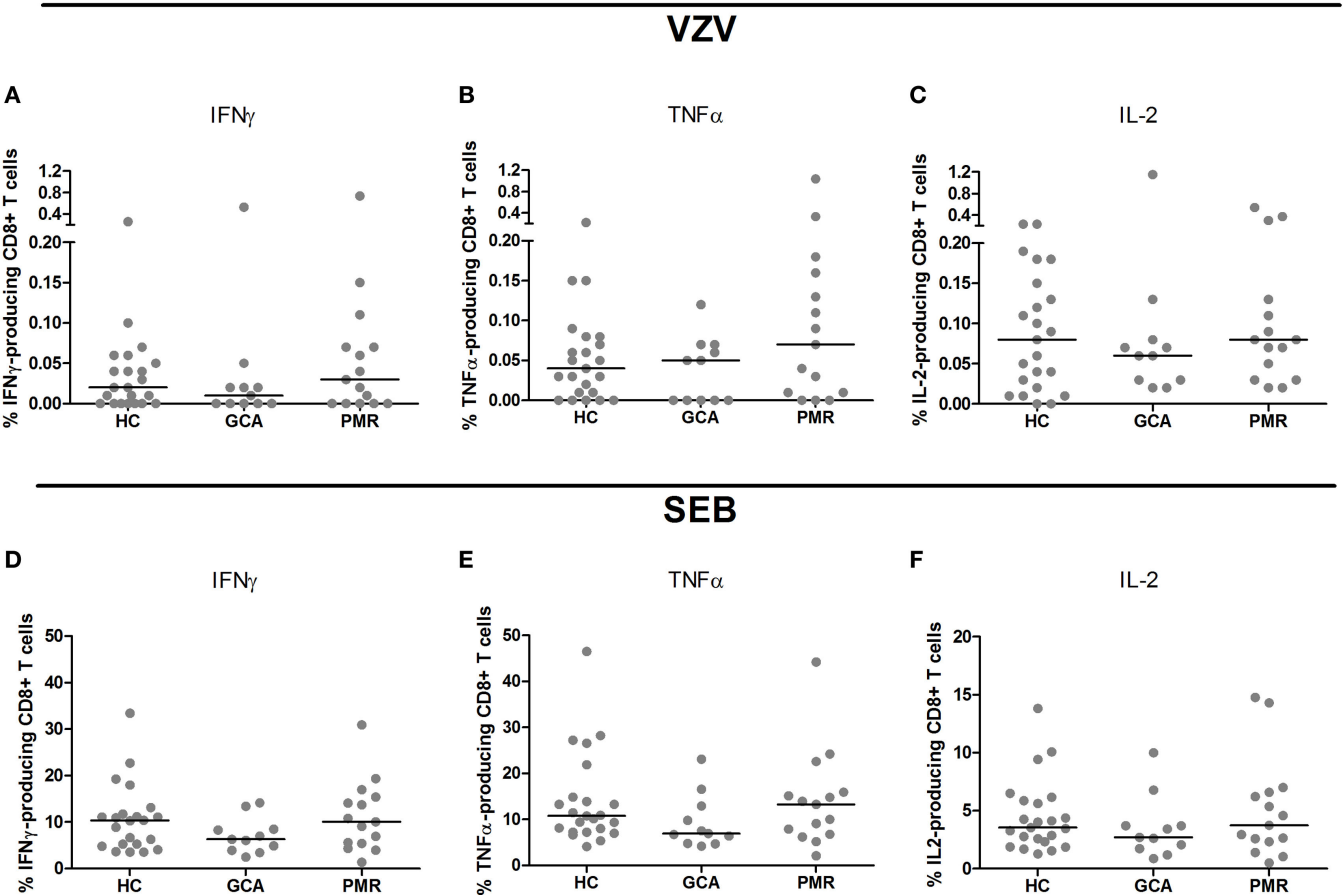
Frequencies of cytokine-producing CD8+ T cells upon stimulation with varicella zoster virus (VZV) **(A–C)** and staphylococcal enterotoxin B (SEB, positive control) **(D–F)** in healthy control (HC) subjects (*n* = 23), giant cell arteritis (GCA) patients (*n* = 11) and polymyalgia rheumatica (PMR) patients (*n* = 15). Results are corrected for responses in non-stimulated cultures. Bars show the median. IFNγ, interferon-γ; TNFα, tumor necrosis factor α; IL-2, interleukin-2.

Upon stimulation with VZV, patients with GCA and HCs showed similar frequencies of cytokine-producing CD4+ T cells. Interestingly, higher frequencies of CD4+ T cells producing IFNγ and IL-2 were found in PMR patients compared to HC subjects (*p* = 0.019 and *p* = 0.048, respectively) (Figures 2A–C). Similar frequencies of cytokine-producing CD8+ T cells were observed in patient groups and controls following stimulation with VZV (Figures 3A–C).

Upon SEB stimulation as positive control for stimulation, patients with GCA, PMR, and control subjects showed similar frequencies of IFNγ-, TNFα, and IL-2-producing CD4+ T cells (Figures 2D–F). For CD8+ T cell cytokine production upon stimulation with SEB there was a trend toward a lower frequency of CD8+ T cells producing TNFα in GCA patients compared to HCs (*p* = 0.077) (Figures 3D–F).

### Humoral Immunity to VZV

**Figure 4:**
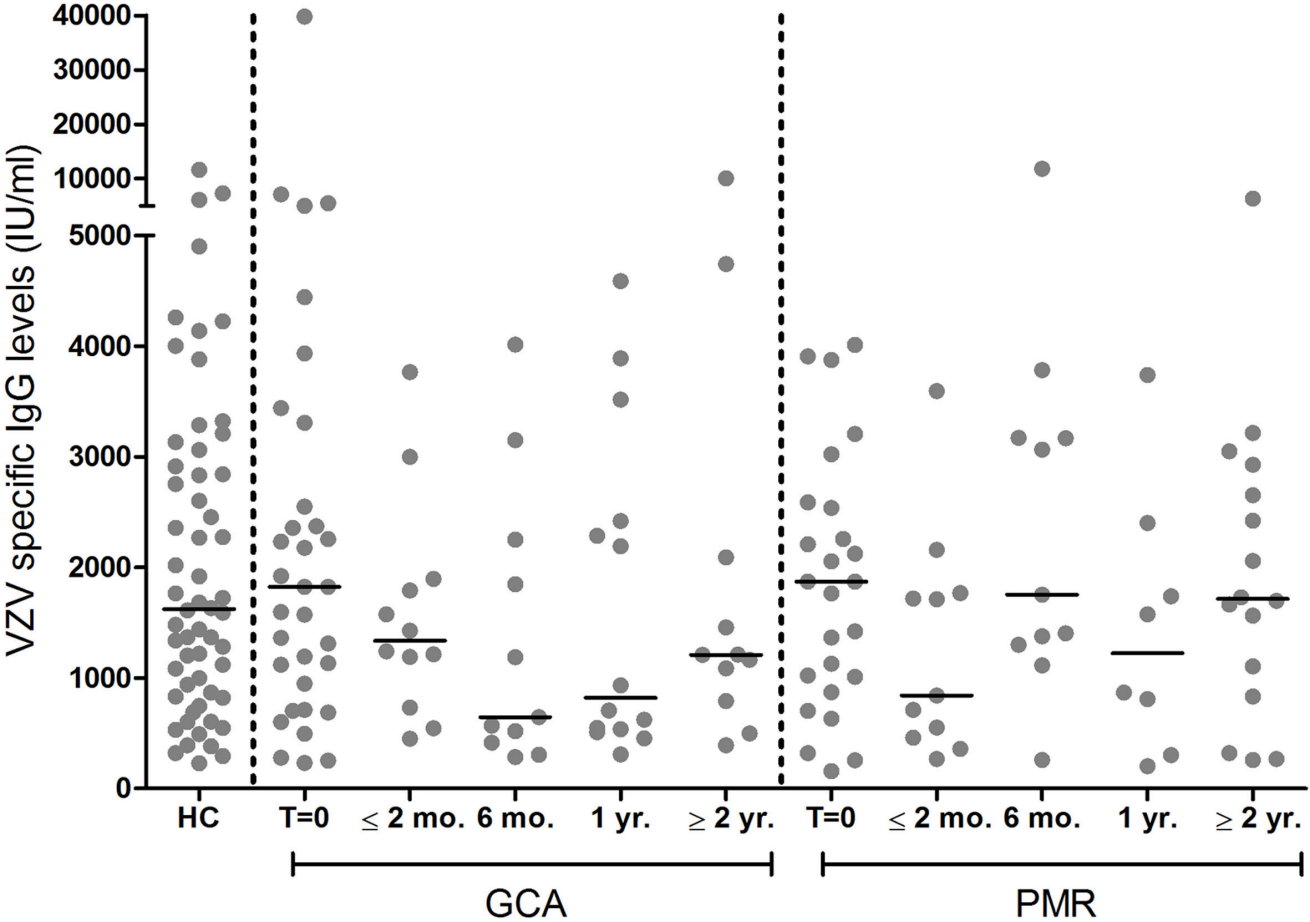
Levels of IgG antibodies against varicella zoster virus (VZV) among 35 giant cell arteritis (GCA) patients and 26 polymyalgia rheumatica (PMR) patients at different time points of follow-up, and 58 healthy control (HC) subjects. Bars show the median. *T* = 0 = before start of treatment with prednisolone; ≤2 months, within 2 months after start of treatment; 6 months, at approximately 6 months of follow-up; 1 year, at approximately 1 year of follow-up; ≥2 year, at approximately 2 years of follow-up, or more.

Median VZV-specific antibody levels of patients at baseline, before starting treatment, were similar in patient groups compared to HCs (*p* = 0.931 for GCA and *p* = 0.777 for PMR, Figure 4). Course of VZV–IgG antibody levels in GCA and PMR patients are shown in Figure 4.

#### Reactivations of VZV

In the group of patients and controls in which VZV-specific IgG levels were determined (Figure 4), one patient was noted to have experienced herpes zoster about 3 months before GCA diagnosis. As can be expected, this patient showed a high VZV-IgG antibody level at time of diagnosis (7,100 IU/ml). In another patient, a very high VZV-IgG antibody level of almost 40,000 IU/ml was found, but medical records did not mention herpes zoster or symptoms suggesting this diagnosis. The high antibody level suggests that this patient is likely to have had a (subclinical) VZV reactivation that escaped diagnosis.

## Discussion

Confirming our hypothesis, a reduced cellular immunity to VZV in GCA patients was demonstrated using IFNγ ELISpot, even when compared to an already at-risk age-matched HC group. Results in patients with PMR were found to be similar compared to age-matched controls. VZV-specific IgG levels were found to be similar when comparing the patient groups to the HC group.

It is widely accepted that cell-mediated immunity is an important measure of susceptibility to VZV (24–26). Since HCs and GCA patients were shown to have similar total numbers of CD3+ T lymphocytes, which probably are the main IFNγ producing cells among PBMCs (27), we can conclude that our results reflect a decreased cell-mediated immunity to VZV. Accordingly, GCA patients are even more prone to herpes zoster than the already at-risk healthy elderly population.

To date only one, for an epidemiological study relatively small, retrospective study investigated the incidence of herpes zoster in GCA patients (with 21 and 38 cases of herpes zoster in 204 GCA patients and 407 controls, respectively). In this study, GCA patients did not seem to be at higher risk of herpes zoster than age-matched controls. However, with an incidence of over 11 per 1,000 person years, the herpes zoster risk in GCA patients was found to be high (28). Interestingly, several large studies reported an increased risk of herpes zoster in persons (mostly diagnosed with rheumatoid arthritis) receiving glucocorticoid therapy (9–14). In our hospital, GCA patients usually start on 40–60 mg of prednisolone daily, while PMR patients start at a lower dose of 15–20 mg prednisolone daily. In both patient groups, the prednisolone dose is slowly tapered.

Because of the high risk, herpes zoster vaccination needs to be considered in this GCA patients. Ideally, vaccination would be administered at time of diagnosis, before patients reach a high cumulative dose of glucocorticoids. Herpes zoster vaccination has been shown to be safe in subjects receiving glucocorticoids, although most subjects studied received relatively low doses (18). Efficacy of herpes zoster vaccination in glucocorticoid treated patients is a topic that needs to be studied further, as only humoral immune responses were evaluated in these patients previously (18).

Due to the limited number of patients included, no firm epidemiologic conclusions can be drawn on the incidence of clinically overt herpes zoster in GCA or PMR patients in the present report. Furthermore, herpes zoster could have been missed before diagnosis and during follow-up, as it does not necessarily come to attention of the treating rheumatology specialist, especially during more stable disease periods in which outpatient clinic visits are less frequent. As mentioned, for one patient notes on a herpes zoster period shortly before GCA disease onset were found in the medical records. In hindsight, another patient possibly had a VZV reactivation at time of GCA diagnosis.

No difference was found in the percentages of cytokine-producing CD4+ and CD8+ T cells in response to VZV stimulation between GCA patients and controls. When evaluating low-level cell-mediated immune responses, ELISpot is considered to be more sensitive than intracellular flow cytometric analysis while cytokine flow cytometry allows for phenotypic discrimination of responding cells (25, 27). The effector function of T cells upon VZV-specific activation is more clearly seen during active secretion of the cytokines, while flow cytometry shows the potential to react to VZV.

Polymyalgia rheumatica patients were shown to have a higher percentage of IL-2 and IFNγ producing CD4+ T cells in response to VZV stimulation using flow cytometry. As the lymphocyte count in PMR patients was lower than in the HC group, the difference in absolute numbers of CD4+ T cells producing cytokines in response to stimulation with VZV between these groups is probably less pronounced than suggested by the relative values.

Of note, as it is known that VZV-specific IgG levels only slowly decline after herpes zoster (29–31), the finding of similar VZV-specific IgG levels in patients at time of diagnosis and HCs, indicates that herpes zoster did not occur substantially more often in GCA and PMR patients in the months before onset of disease.

In recent years, several studies have investigated the link between VZV and GCA with the finding of VZV antigen in temporal artery biopsies of a majority of GCA patients as the pivotal evidence claiming an etiologic role for VZV in GCA (32–34). Other research groups, however, did not detect VZV antigen nor VZV-DNA in temporal arteries of GCA patients (35–37). A recent paper by Rhee et al., describing a large retrospective study using an electronic database, reported an only modest association between antecedent infections and the incidence of GCA. GCA patients were more likely to have had a prior herpes zoster infection (median time from zoster to GCA of 6 years) than control subjects, but also had significantly more other infections, e.g., respiratory and urinary tract infections. No higher occurrence of clinically overt herpes zoster infections at time of diagnosis was found (38). Our results, demonstrating similar humoral immunity in GCA and PMR patients when compared to HCs, are in line with these results.

To the best of our knowledge, this is the first prospective study investigating both humoral and cellular immunity to VZV in GCA and PMR patients. Concluding with our most important result, a decreased cell-mediated immunity to VZV indicates an even higher risk to herpes zoster in GCA patients on glucocorticoid treatment than in age-matched HCs. Herpes zoster vaccination, therefore, needs to be considered in patients with GCA and should ideally be administered at time of diagnosis, before reaching high cumulative doses of glucocorticoids.

## Ethics Statement

All subjects gave written informed consent in accordance with the Declaration of Helsinki. The study was approved by the institutional review board of the University Medical Centre Groningen (METc2012/375 for healthy controls and METc2010/222 for GCA and PMR patients).

## Author Contributions

All authors contributed to the design and discussion of the project. Acquisition of data was carried out by CR, KG, EE, and EB. CR analyzed data and drafted the manuscript. EE conducted most of the experiments and helped with analysis. All authors revised the article critically and approved the final version of the manuscript.

## Conflict of Interest Statement

AB has received consulting fees from Grunenthal GmbH. No conflict of interest has been declared by the other authors.

## Supplementary Material

Table S1 Supplementary Table S1 presents characteristics of all patients in whom VZVIgG antibody levels were measured per follow-up time point (Figure 4).

## Abbreviations

GCA, giant cell arteritis; PBMC, peripheral blood mononuclear cell; PMR, polymyalgia rheumatica; SEB, staphylococcal enterotoxin B; VZV, varicella zoster virus.

## References

[1] Heininger U Seward JF. Varicella. Lancet (2006) 368:1365–76. 10.1016/S0140-6736(06)69561-5 17046469

[2] Bond D Mooney J. A literature review regarding the management of varicella-zoster virus. Musculoskeletal Care (2010) 8:118–22. 10.1002/msc.175 20301227

[3] Drolet M Brisson M Schmader KE Levin MJ Johnson R Oxman MN The impact of herpes zoster and postherpetic neuralgia on health-related quality of life: a prospective study. CMAJ (2010) 182:1731–6. 10.1503/cmaj.091711 20921251 PMC2972323

[4] Drolet M Levin MJ Schmader KE Johnson R Oxman MN Patrick D Employment related productivity loss associated with herpes zoster and postherpetic neuralgia: a 6-month prospective study. Vaccine (2012) 30:2047–50. 10.1016/j.vaccine.2012.01.045 22285632

[5] Yawn BP Saddier P Wollan PC St. Sauver JL Kurland MJ Sy LS. A population-based study of the incidence and complication rates of herpes zoster before zoster vaccine introduction. Mayo Clin Proc (2007) 82:1341–9. 10.4065/82.11.1341 17976353

[6] Oxman MN Levin MJ Johnson GR Schmader KE Straus SE Gelb LD A vaccine to prevent herpes zoster and postherpetic neuralgia in older adults. N Engl J Med (2005) 352:2271–84. 10.1056/NEJMoa051016 15930418

[7] Johnson RW Alvarez-Pasquin MJ Bijl M Franco E Gaillat J Clara JG Herpes zoster epidemiology, management, and disease and economic burden in Europe: a multidisciplinary perspective. Ther Adv Vaccines (2015) 3:109–20. 10.1177/2051013615599151 26478818 PMC4591524

[8] Dejaco C Duftner C Buttgereit F Matteson EL Dasgupta B. The spectrum of giant cell arteritis and polymyalgia rheumatica: revisiting the concept of the disease. Rheumatology (Oxford) (2017) 56:506–15. 10.1093/rheumatology/kew273 27481272

[9] Pappas DA Hooper MM Kremer JM Reed G Shan Y Wenkert D Herpes zoster reactivation in patients with rheumatoid arthritis: analysis of disease characteristics and disease-modifying antirheumatic drugs. Arthritis Care Res (Hoboken) (2015) 67:1671–8. 10.1002/acr.2262 26018115

[10] Zhang J Xie F Delzell E Chen L Winthrop KL Lewis JD Association between vaccination for herpes zoster and risk of herpes zoster infection among older patients with selected immune-mediated diseases. JAMA (2012) 308:43–9. 10.1001/jama.2012.7304 22760290 PMC3683869

[11] Smitten AL Choi HK Hochberg MC Suissa S Simon TA Testa MA The risk of herpes zoster in patients with rheumatoid arthritis in the United States and the United Kingdom. Arthritis Rheum (2007) 57:1431–8. 10.1002/art.23112 18050184

[12] Strangfeld A Listing J Herzer P Liebhaber A Rockwitz K Richter C Risk of herpes zoster in patients with rheumatoid arthritis treated with anti-TNF-alpha agents. JAMA (2009) 301:737–44. 10.1001/jama.2009.146 19224750

[13] Winthrop KL Baddley JW Chen L Liu L Grijalva CG Delzell E Association between the initiation of anti-tumor necrosis factor therapy and the risk of herpes zoster. JAMA (2013) 309:887–95. 10.1001/jama.2013.1099 23462785 PMC3773213

[14] Westra J Rondaan C van Assen S Bijl M Vaccination of patients with autoimmune inflammatory rheumatic diseases. Nat Rev Rheumatol (2015) 11:135–45. 10.1038/nrrheum.2014.206 25486980

[15] Kimberlin DW Whitley RJ Varicella-zoster vaccine for the prevention of herpes zoster. N Engl J Med (2007) 356:1338–43. 10.1056/NEJMct066061 17392303

[16] Harpaz R Ortega-Sanchez IR Seward JF Advisory Committee on Immunization Practices (ACIP) Centers for Disease Control and Prevention (CDC). Prevention of herpes zoster: recommendations of the Advisory Committee on Immunization Practices (ACIP). MMWR Recomm Rep (2008) 57:CE2–4. 18528318

[17] van Assen S Agmon-Levin N Elkayam O Cervera R Doran MF Dougados M EULAR recommendations for vaccination in adult patients with autoimmune inflammatory rheumatic diseases. Ann Rheum Dis (2011) 70:414–22. 10.1136/ard.2010.137216 21131643

[18] Russell AF Parrino J Fisher CL Jr Spieler W Stek JE Coll KE Safety, tolerability, and immunogenicity of zoster vaccine in subjects on chronic/maintenance corticosteroids. Vaccine (2015) 33:3129–34. 10.1016/j.vaccine.2015.04.090 25964168

[19] van der Geest KS Abdulahad WH Chalan P Rutgers A Horst G Huitema MG Disturbed B cell homeostasis in newly diagnosed giant cell arteritis and polymyalgia rheumatica. Arthritis Rheumatol (2014) 66:1927–38. 10.1002/art.38625 24623536

[20] van der Geest KS Abdulahad WH Rutgers A Horst G Bijzet J Arends S Serum markers associated with disease activity in giant cell arteritis and polymyalgia rheumatica. Rheumatology (Oxford) (2015) 54:1397–402. 10.1093/rheumatology/keu526 25724206

[21] Chuang TY Hunder GG Ilstrup DM Kurland LT. Polymyalgia rheumatica: a 10-year epidemiologic and clinical study. Ann Intern Med (1982) 97:672–80. 10.7326/0003-4819-97-5-672 6982645

[22] Rondaan C de Haan A Horst G Hempel JC van Leer C Bos NA Altered cellular and humoral immunity to varicella-zoster virus in patients with autoimmune diseases. Arthritis Rheumatol (2014) 66:3122–8. 10.1002/art.38804 25223407

[23] Holvast A van Assen S de Haan A Huckriede A Benne CA Westra J Studies of cell-mediated immune responses to influenza vaccination in systemic lupus erythematosus. Arthritis Rheum (2009) 60:2438–47. 10.1002/art.24679 19644961

[24] Shirane R Tang H Hayashi K Okuno Y Iso H Asada H Relationship between cell-mediated immunity to Varicella-Zoster virus and aging in subjects from the community-based Shozu Herpes Zoster study. J Med Virol (2017) 89:313–7. 10.1002/jmv.24629 27420414

[25] Levin MJ Smith JG Kaufhold RM Barber D Hayward AR Chan CY Decline in varicella-zoster virus (VZV)-specific cell-mediated immunity with increasing age and boosting with a high-dose VZV vaccine. J Infect Dis (2003) 188:1336–44. 10.1086/379048 14593591

[26] Gershon AA Gershon MD. Pathogenesis and current approaches to control of varicella-zoster virus infections. Clin Microbiol Rev (2013) 26:728–43. 10.1128/CMR.00052-13 24092852 PMC3811230

[27] Karlsson AC Martin JN Younger SR Bredt BM Epling L Ronquillo R Comparison of the ELISPOT and cytokine flow cytometry assays for the enumeration of antigen-specific T cells. J Immunol Methods (2003) 283:141–53. 10.1016/j.jim.2003.09.001 14659906

[28] Schafer VS Kermani TA Crowson CS Hunder GG Gabriel SE Ytterberg SR Incidence of herpes zoster in patients with giant cell arteritis: a population-based cohort study. Rheumatology (Oxford) (2010) 49:2104–8. 10.1093/rheumatology/keq200 20627970 PMC2981027

[29] Cradock-Watson JE Ridehalgh MK Bourne MS Specific immunoglobulin responses after varicella and herpes zoster. J Hyg (Lond) (1979) 82:319–36. 10.1017/S0022172400025730 219110 PMC2130148

[30] Weinberg A Zhang JH Oxman MN Johnson GR Hayward AR Caulfield MJ Varicella-zoster virus-specific immune responses to herpes zoster in elderly participants in a trial of a clinically effective zoster vaccine. J Infect Dis (2009) 200:1068–77. 10.1086/605611 19712037 PMC4014851

[31] Schub D Janssen E Leyking S Sester U Assmann G Hennes P Altered phenotype and functionality of varicella zoster virus-specific cellular immunity in individuals with active infection. J Infect Dis (2015) 211:600–12. 10.1093/infdis/jiu500 25180236

[32] Mitchell BM Font RL. Detection of varicella zoster virus DNA in some patients with giant cell arteritis. Invest Ophthalmol Vis Sci (2001) 42:2572–7. 11581201

[33] Nagel MA Khmeleva N Boyer PJ Choe A Bert R Gilden D. Varicella zoster virus in the temporal artery of a patient with giant cell arteritis. J Neurol Sci (2013) 335:228–30. 10.1016/j.jns.2013.09.034 24125020 PMC3848200

[34] Nagel MA White T Khmeleva N Rempel A Boyer PJ Bennett JL Analysis of varicella-zoster virus in temporal arteries biopsy positive and negative for giant cell arteritis. JAMA Neurol (2015) 72:1281–7. 10.1001/jamaneurol.2015.2101 26349037 PMC5110206

[35] Kennedy PG Grinfeld E Esiri MM. Absence of detection of varicella-zoster virus DNA in temporal artery biopsies obtained from patients with giant cell arteritis. J Neurol Sci (2003) 215:27–9. 10.1016/S0022-510X(03)00167-9 14568124

[36] Bhatt AS Manzo VE Pedamallu CS Duke F Cai D Bienfang DC In search of a candidate pathogen for giant cell arteritis: sequencing-based characterization of the giant cell arteritis microbiome. Arthritis Rheumatol (2014) 66:1939–44. 10.1002/art.38631 24644069 PMC4113339

[37] Alvarez-Lafuente R Fernandez-Gutierrez B Jover JA Judez E Loza E Clemente D Human parvovirus B19, varicella zoster virus, and human herpes virus 6 in temporal artery biopsy specimens of patients with giant cell arteritis: analysis with quantitative real time polymerase chain reaction. Ann Rheum Dis (2005) 64:780–2. 10.1136/ard.2004.025320 15834059 PMC1755502

[38] Rhee RL Grayson PC Merkel PA Tomasson G Infections and the risk of incident giant cell arteritis: a population-based, case-control study. Ann Rheum Dis (2017) 76:1031–35. 10.1136/annrheumdis-2016-210152 27895041 PMC12359076

