# Supplementary material for: Decreased Immunity to Varicella Zoster Virus in Giant Cell Arteritis
Source: Front Immunol. 2017 Oct 24;8:1377. doi: 10.3389/fimmu.2017.01377 (PMC5661055; doi:10.3389/fimmu.2017.01377)
Supplement: Table S1 — Supplementary Table S1 presents characteristics of all patients in whom VZVIgG antibody levels were measured per follow-up time point (Figure 4). [file table_1.pdf]

Supplementary Table 1. Characteristics of patients and healthy controls whose samples were used for determination of VZV-IgG levels (Figure 4).

	**HC (n=58)**	**GCA patients (n=35)**					**PMR patients (n=26)**				
Sex, no. female/male	41/17	25/10					17/9				
Age at time of first sample, median (interquartile range) years	67.7 (62.2-74.9)	73.2 (63.8-79.0)					73.7 (64.7-78.9) *				
GCA diagnosis at baseline: FDG PET-CT|TAB|FDG PET-CT+TAB	NA	17|9|9					NA				
PMR diagnosis at baseline: FDG PET-CT|Chuang|FDG PET-CT+Chuang	NA	3|0|6					3|5|17†				
		**T=0** (n=33)	**≤2 mo.** (n=12)	**6 mo.** (n=11)	**1 yr.** (n=14)	**≥2 yr.** (n=11)	**T=0** (n=25)	**≤2 mo.** (n=12)	**6 mo.** (n=11)	**1 yr.** (n=8)	**≥2 yr.** (n=16)
Time since diagnosis, median (interquartile range) months		NA	0.5 (0.4-0.7)	7.1 (6.4-8.6)	11.6 (10.2-13.6)	32.6 (21.3-35.8)	NA	0.7 (0.5-0.8)	7.5 (6.8-8.4)	9.8 (9.4-10.5)	24.9 (23.0-27.8)
Cumulative prednisone dosage, median (interquartile range) mg	NA	NA‡	840 (615-1185)	5655 (5600-6233)	6561 (5600-8175)	10808 (7446-12186)	NA	360 (320-1670)	2760 (1895-3090)	3375 (3198-4301)	5949 (3377-7600)
Use of methotrexate, no. (%) – dose range in mg/week	NA	NA	0 (0)	1 (9) – 15	2 (14) – 15-15	3 (27) – 15-25	NA	0 (0)	0 (0)	1 (13) - 15	1 (6) - 10
Use of leflunomide, no. (%) – dose range in mg/day	NA	NA	0 (0)	0 (0)	1 (7) - 10	0 (0)	NA	0 (0)	1 (9) - 10	0 (0)	1 (6) - 10
No. of patients that experienced relapse since diagnosis (%)	NA	NA	1 (9)	0 (0)	6 (43)	6 (55)	NA	0 (0)	4 (36)	2 (25)	9 (56)
Leukocyte count, median (interquartile range) 10^9 cells/L	5.5 (4.9-6.4)	9.1 (8.1-11.5) ***	17.2 (9.2-23.6) ***	11 (9.6-12.2) ***	9.5 (7.2-12.2) ***	7.9 (7.0-9.8) ***	8.7 (7.1-11.1) ***	11.4 (9.3-13.5) ***	9.1 (6.2-12.3) ***	8.3 (7.5-9.3) ***	7.4 (5.5-9.7) **
Haemoglobin, median (interquartile range) mmol/l	8.7 (8.2-9.2)	7.1 (6.6-7.5) ***	7.8 (7.3-8.0) ***	8.1 (7.5-8.5) **	8.1 (6.6-8.8) **	7.7 (6.8-8.3) ***	7.5 (7.3-8.4) ***	8.3 (7.6-8.7) *	8.1 (7.8-9.2)	8.4 (7.8-9.2) *	8.2 (7.1-9.0)
ESR, median (interquartile range) mm/hr	9 (4-13)	99 (59-104) ***	11 (5-45)	24 (16-34) ***	28 (14-37) ***	28 (15-40) ***	51 (39-74) ***	12 (6-30)	22 (9-30) **	13 (3-44)	15 (5-36)
CRP, median (interquartile range) mg/L	<5 (<5-<5)	54 (25-96) ***	5 (<5-6) ***	7 (<5-12) ***	6 (<5-13) ***	10 (<5-15) ***	44 (27-73) ***	<5 (<5-7) **	7 (<5-11) ***	10 (<5-26) ***	<5 (<5-6) **

HC = healthy controls; GCA = giant cell arteritis; PMR = polymyalgia rheumatica; T=0 = before start of treatment with prednisone; ≤ 2 mo. = within 2 months after start of treatment; 6 mo. = at approximately 6 months of follow-up; 1 yr. = at approximately 1 year of follow-up; 2 yr. = at approximately 2 years of follow-up, or more; FDG PET-CT = no. of patients with ^{18}F-fluorodeoxyglucose-positron emission tomography/computed tomography supporting diagnosis; TAB = no. of patients with temporal artery biopsy positive for GCA; NA = not applicable; Chuang: no. of patients fulfilling the Chuang and Hunder PMR criteria; ESR = erythrocyte sedimentation rate; CRP = C-reactive protein.

† One patient did not fulfil Chuang and Hunder criteria or had FDG PET-CT proven PMR, but fulfilled the 2012 European League Against Rheumatism/American College of Rheumatology criteria for PMR.

‡ One patient received prednisone treatment because of PMR until approximately 5 months before GCA diagnosis.

* P value <0.05; ** P value <0.01; *** P value <0.001 for comparison to healthy control group.
